# Exploring Health Literacy in Medical University Students of Chongqing, China: A Cross-Sectional Study

**DOI:** 10.1371/journal.pone.0152547

**Published:** 2016-04-06

**Authors:** Yan Zhang, Fan Zhang, Ping Hu, Wenjie Huang, Lu Lu, Ruixue Bai, Manoj Sharma, Yong Zhao

**Affiliations:** 1 School of Public Health and Management, Chongqing Medical University, Chongqing, China; 2 Research Center for Medicine and Social Development, Chongqing Medical University, Chongqing, China; 3 Innovation Center for Social Risk Governance in Health, Chongqing Medical University, Chongqing, China; 4 Department of Behavioral and Environmental Health, Jackson State University, Jackson, United States of America; Iranian Institute for Health Sciences Research, ACECR, ISLAMIC REPUBLIC OF IRAN

## Abstract

Health literacy is important in public health and healthcare, particularly in effective communication between patients and health professionals. Although most medical students will eventually work as health professionals after graduation, research on health literacy of medical students is scarce. This study aimed to assess the health literacy level of medical students in Chongqing, China, and its influencing factors. A cross-sectional study was conducted and 1,275 participants (250 males and 1,022 females) who majored in five different disciplines were involved. The Health Literacy Questionnaire was used as the survey tool. The junior students obtained the highest scores, whereas the freshman students had the lowest scores on each scale. The average score of males was higher than that of females except in “feeling understood and supported by healthcare providers,” and the average score of students who reside in urban areas was higher than that of students in rural areas. Moreover, the average score of engineering students was higher than that of medical or health sciences students. Multiple linear regression models (R_adj_^2^ = 0.435, P = 0.000) showed that the grade, socioeconomic status, and parent’s highest level of education were positively correlated with health literacy. In conclusion, the health literacy levels of the medical students are insufficient and need improvement.

## Introduction

The term health literacy was first used in the 1970s [1] and has become increasingly important in public health and healthcare [2]. However, unified standard definition for this term has not been established; health literacy is often defined as the ability to obtain, process, and understand basic health information and services to make appropriate health decisions [3]. Some studies have proven that individual health literacy levels would have far-reaching influence on individuals and the society [4], and inadequate health literacy is an independent risk factor for hospital admission [5]. Inadequate health literacy is connected with insufficient understanding of written information and poor communication with healthcare professionals [6–8]. A survey showed that in 2003, more than one third of Americans had low health literacy [9]; in 2006, the Australian Bureau of Statistics found that nearly 60% of adult Australians had low health literacy [10]. In 2012, only 8.80% Chinese residents had basic health literacy [11]. The questionnaire developed from the evaluation index system of Chinese health literacy included basic health knowledge and concept, healthy lifestyle and behavior, and basic skills.

Health literacy is crucial in the effective communication between patients and health professionals, because the latter can help enhance the health literacy of the former [12]. However, studies on the health literacy of health professionals are limited [13–15]. International research suggests that significant gaps in awareness, knowledge, and clinical recognition of low health literacy exist among health professionals [16]. Most medical students will work as health professionals after graduation and will have more opportunities to interact with patients. Therefore, their health literacy levels need more attention. Many countries have realized the importance of health literacy but have focused on patients, residents, and non-medical students. Limited sources on this topic showed that even health professionals and students in the United States lack health literacy [16]. A cross-sectional study on health-related knowledge among Chinese vocational college students showed that the level of health literacy in medical major students was inadequate [17].

Three of the most widely used measures of health literacy are (1) the Newest Vital Sign (a short clinical screening tool) [18], (2) the Rapid Estimate of Adult Literacy in Medicine [19], and (3) the Test of Functional Health Literacy in Adults [20]. However, these measures do not reflect the full definition of health literacy and have psychometric weaknesses [21]. In the present study, we used the health literacy questionnaire (HLQ) created by Osborne et al. to identify the specific health literacy strengths and limitations of participants [22]. The initial validation study of HLQ was set in several medical institutions, such as hospitals and private clinics [22]. The HLQ has strong construct validity, reliability, and high acceptability [22]. Currently, the HLQ is being validated in medical students in several universities, such as New Zealand, Australia, Hong Kong, and the United Kingdom. HLQ has not been previously used in Mainland China.

We aimed to assess the health literacy of medical university students in Chongqing, China. The influencing factors of health literacy among medical students were also investigated.

## Methods

### Study design

A cross-sectional study was conducted involving undergraduates of a medical university in Chongqing, China. Stratified cluster sampling was adopted, and the grade was used as the primary sampling unit. We chose three grades, namely, Grades One, Two, and Three. Then, we randomly selected five different disciplines, namely dietetics, nursing, physiotherapy, preventive medicine, and biomedical engineering. Finally, all the students from these disciplines participated in this investigation.

### Ethical approval

This study was approved by the Ethics Committee of Chongqing Medical University, China (Preference number: 2015002). Written informed consent was obtained from all participants.

### Sample

In this study, we distributed 1,332 questionnaires. A total of 1,275 questionnaires were recovered, and the response rate was 95.72%. Three responses were deleted because of missing data, which resulted in a final sample of 1,272 in the analysis.

### Questionnaire

HLQ was developed by Osborne et al. [22] and has been validated with excellent psychometric properties, construct validity, reliability, and high acceptability [22, 23]. The HLQ was translated into several languages for non-English-speaking participants, including Chinese, Greek, Italian, and Vietnamese [24]. In the current study, we used the existing Chinese version of HLQ, which contains three parts. Part 1 is about demographic characteristics, including sex, age, type of residence, socioeconomic status, parent’s highest level of education, long-term illness or disability, faculty, majors, and grade. Parts 2 and 3 contain 44 questions across nine domains: 1) feeling understood and supported by healthcare providers; 2) having sufficient information to manage my health; 3) actively managing my health; 4) social support for health; 5) appraisal of health information; 6) ability to actively engage with healthcare providers; 7) navigating the healthcare system; 8) ability to find good health information; and 9) understanding health information well enough to know what to do. In addition, scales 1 to 5 were about how strongly the participant disagree or agree with the statements, whereas scales 6 to 9 were about how easy or difficult the tasks are. Every domain contained 4 to 6 questions.

### Survey implementation

Before the formal investigation, we conducted a pre-survey involving 20 medical students in the same university. We re-examined its reliability by assessing the internal reliability and the Cronbach’s α = 0.947 after performing the pre-survey. In the formal investigation, a class served as the survey unit. We contacted the monitors and the classroom teachers in advance to ensure their support and understanding. Before filling in the questionnaire, the teacher and the monitor organized the students. The investigators gave a simple introduction about the project and obtained the consent of the students. The HLQ was filled in during recess because every questionnaire required 10–15 min to complete.

### Data analyses

The data were carefully checked before entering the database, which was established using the Epi-data 2.1 software. The data were meticulously sorted, cleaned, and analyzed using SPSS 17.0. The algorithm produced unweighted scale scores for the nine dimensions of HLQ, and the final score for each subscale was an average score across all items forming the scale. Score ranged 1–4 for scales 1–5 and 1–5 for scales 6–9. This program used the EM algorithm to impute missing values. Scales with 4–5 items allowed two missing values to be imputed. Scales with six items allowed for three missing values to be imputed. If more responses among the scales items were missing, the scale score would not be computed.

The points set were as follows: strongly disagree = 1, disagree = 2, agree = 3, strongly agree = 4; cannot do = 1, very difficult = 2, quite difficult = 3, quite easy = 4, very easy = 5. Scales 1 and 2 included four subjects; hence, the full marks were 16 points. Scales 3 to 5 included five subjects; hence, the full marks were 20 points. Scales 6, 8, and 9 included five subjects; hence, the full marks were 25 points. Scale 7 included six subjects; hence, the full mark was 30 points. We set the low health literacy to scores less than 60% total score and the high health literacy to scores more than 80% total score.

We set up multiple linear regression models. The age, sex, type of residence, socioeconomic status, parent’s highest level of education, long-standing illness, faculty, and grade were incorporated into the models. The method of “enter” was used to eliminate variables.

Chi-square test, ANOVA, T- test, multiple linear regression models, and enter method were used. Statistical significance was defined as p-value <0.05.

## Results

### Characteristics of Participants

As shown in Table 1, the participants included 250 (19.7%) males and 1,022 (80.3%) females. Specifically, 43.9% of the participants were freshmen students, 31.5% were sophomore students, and 24.5% were junior students; 56.7% majored in nursing, 17.1% in preventive medicine, 10.6% in physiotherapy, 8.6% in dietetics, and 7.0% in biomedical engineering.

**Table 1:** Demographic Characteristics of Different Grades.

Demographic characteristics	Total (N = 1272)	Grades			P-value
		Grade One (N = 559)	Grade Two (N = 401)	Grade Three (N = 312)	
Age					0.000
15–19 years old	508 (39.9%)	409 (73.2%)	94 (23.4%)	5 (1.6%)	
20–24 years old	762 (59.9%)	150 (26.8%)	306 (76.3%)	306 (98.1%)	
25–29 years old	1 (0.1%)	0 (0.0%)	0 (0.0%)	1 (0.3%)	
More Than 30 years old	1 (0.1%)	0 (0.0%)	1 (0.2%)	0 (0.0%)	
Sex					0.392
Male	250 (19.7%)	101 (18.1%)	81 (20.2%)	68 (21.8%)	
Female	1022 (80.3%)	458 (81.9%)	320 (79.8%)	244 (78.2%)	
Type of residence					0.000
Urban area	670 (52.7%)	258 (46.2%)	232 (57.9%)	180 (57.7%)	
Rural area	602 (47.3%)	301 (53.8%)	169 (42.1%)	132 (42.3%)	
Socioeconomic status					0.001
Below average	497 (39.1%)	227 (40.6%)	134 (33.4%)	136 (43.6%)	
Average	622 (48.9%)	262 (46.9%)	216 (53.9%)	144 (46.2%)	
Above average	27 (2.1%)	6 (1.1%)	17 (4.2%)	4 (1.3%)	
I do not know	126 (9.9%)	64 (11.4%)	34 (8.5%)	28 (9.0%)	
Parent’s highest level of education					0.004
Has not completed high school/secondary school	721 (56.7%)	326 (58.3%)	211 (52.6%)	184 (59.0%)	
Completed high school/secondary school	305 (24.0%)	135 (24.2%)	100 (24.9%)	70 (22.4%)	
Attained a diploma or certificate from a tertiary institution	102 (8.0%)	34 (6.1%)	37 (9.2%)	31 (9.9%)	
Attained a bachelor’s degree from a tertiary institution	92 (7.2%)	45 (8.1%)	27 (6.7%)	20 (6.4%)	
Attained a master’s degree from a tertiary institution	10 (0.8%)	1 (0.2%)	8 (2.0%)	1 (0.3%)	
Attained a PhD from a tertiary institution	1 (0.1%)	1 (0.2%)	0 (0.0%)	0 (0.0%)	
Question does not apply to me	10 (0.8%)	1 (0.2%)	8 (2.0%)	1 (0.3%)	
I do not know	31 (2.4%)	16 (2.9%)	10 (2.5%)	5 (1.6%)	
What faculty are you currently enrolled in					0.527
Engineering	89 (7.0%)	34 (6.1%)	31 (7.7%)	24 (7.7%)	
Medical or health sciences	1183 (93.0%)	525 (93.9%)	370 (92.3%)	288 (92.3%)	
Which type of program are you studying					0.000
Biomedical engineering	89 (7.0%)	34 (6.1%)	31 (7.7%)	24 (7.7%)	
Dietetics	109 (8.6%)	37 (6.6%)	34 (8.5%)	38 (12.2%)	
Nursing	721 (56.7%)	366 (65.5%)	205 (51.1%)	150 (48.1%)	
Physiotherapy	135 (10.6%)	52 (9.3%)	44 (11.0%)	39 (12.5%)	
Preventive medicine	218 (17.1%)	70 (12.5%)	87 (21.7%)	61 (19.6%)	

In addition, 52.7% of the participants came from urban areas, whereas 47.3% of the participants came from rural areas; 88.0% of the participants considered their socioeconomic status to be below average or average, and 80.7% of the participants indicated that their parents completed high school/secondary school or lower. Of the participants, 3.3% had depression or anxiety, whereas 91% had no long-standing illness.

#### Demographic characteristics of different grades

Three groups were classified according to their grades, namely Grades One, Two, and Three. Large differences in the proportion of males and females are observed in Table 1. The number of females (80%) far exceeded that of males in every grade. Most of the participants in each grade thought that their socioeconomic status was below average or average, and more than half in every grade said that their parents had not completed high school/secondary school. We found that age (p = 0.000), type of residence (p = 0.000), socioeconomic status (p = 0.001), parent’s highest level of education (p = 0.004), and disciplines (p = 0.000) significantly varied in different grades.

### HLQ scores

Overall, the total score of HLQ was 197.00; the mean score of HLQ was 131.89 (SD: 18.84) and this was equivalent to 66.9% of the total score. We found that 20.4% of the participants had low health literacy and only 5.7% of the participants had high health literacy. Specifically speaking, the average scores of nine scales were as follows (from scales 1 to 9): 8.59±2.32, 10.18±1.92, 13.10±2.40, 14.59±2.25, 13.12±2.33, 16.73±3.63, 19.58±4.11, 17.61±3.15, and 18.38±2.97.

The lowest score was recorded in “feeling understood and supported by healthcare providers,” and the highest score was in “understand health information well enough to know what to do.”

#### HLQ score in different grades

Table 2 shows that, in each scale, the scores in Grade Three were highest, whereas the scores in Grade One were lowest. The difference was statistically significant (P = 0.000). In “feeling understood and supported by healthcare providers,” “actively managing my health,” “appraisal of health information,” and “ability to actively engage with healthcare providers,” the average scores in Grades One and Two were lower than the total average scores were. In “having sufficient information to manage my health,” “social support for health,” “navigating the healthcare system,” “ability to find good health information,” and “understand health information well enough to know what to do,” only the average scores in Grade One were lower than the total average scores were. The score difference in every scale between Grades One and Two, Grades One and Three, and Grades Two and Three were statistically significant (P<0.01).

**Table 2:** Average Score of Each Scale in Different Grades, Sex, Type of Residence, and Faculties (M±SD).

	Scale 1	Scale 2	Scale 3	Scale 4	Scale 5	Scale 6	Scale 7	Scale 8	Scale 9
Grades									
Grade One	8.08±2.25	9.64±1.92	12.55±2.50	13.90±2.30	12.49±2.36	14.23±3.44	17.09±3.83	15.79±3.27	16.44±3.01
Grade Two	8.48±1.94	10.19±1.54	13.09±1.82	14.68±1.81	13.04±0.36	18.01±1.98	20.30±2.57	18.32±1.79	19.09±1.47
Grade Three	9.65±2.54	11.15±2.00	14.08±2.57	15.72±2.22	14.35±2.38	19.59±2.44	23.11±3.15	19.97±2.23	20.96±1.75
Sex									
Male	8.53±2.39	10.37±2.02	13.35±2.47	14.73±2.18	13.24±2.34	17.22±3.49^a^	20.10±4.10^b^	18.07±3.13^c^	18.84±2.92^d^
Female	8.60±2.31	10.14±1.90	13.03±2.38	14.56±2.27	13.09±2.32	16.61±3.65^a^	19.45±4.10^b^	17.50±3.14^c^	18.27±2.97^d^
Type of residence									
Urban area	8.61±2.33	10.29±2.00^e^	13.13±2.53	14.72±2.24^f^	13.20±2.41	17.04±3.45^g^	19.93±4.15^h^	17.81±3.13^i^	18.73±2.95^j^
Rural area	8.57±2.31	10.06±1.83^e^	13.06±2.25	14.45±2.26^f^	13.03±2.22	16.39±3.80^g^	19.18±4.04^h^	17.39±3.15^i^	18.00±2.96^j^
Faculty									
Engineering	9.62±2.62^k^	11.12±2.25^l^	13.94±2.81^m^	15.31±2.37^n^	13.92±2.83^o^	18.92±3.57^p^	22.63±3.62^q^	19.45±3.02^r^	20.12±2.38^s^
Medical or health sciences	8.51±2.28^k^	10.11±1.88^l^	13.03±2.36^m^	14.54±2.23^n^	13.06±2.27^o^	16.57±3.58^p^	19.35±4.05^q^	17.48±3.12^r^	18.25±2.97^s^

Scale 1: Feeling understood and supported by healthcare providers; Scale 2: Having sufficient information to manage my health; Scale 3: Actively managing my health; Scale 4: Social support for health; Scale 5: Appraisal of health information; Scale 6: Ability to actively engage with healthcare providers; Scale 7: Navigating the healthcare system; Scale 8: Ability to find good health information; Scale 9: Understand health information well enough to know what to do.

^a, b, c, d, e, f, g, h, i, j, k, l, m, n, o, p, q, r, s^ Values with the same superscripts are significantly different by age group at p < 0.05 (Using T-test).

#### Average score of each scale in different sex

In every scale, the average scores in males were higher than those in females were, except in “feeling understood and supported by healthcare providers.” The differences in “ability to actively engage with healthcare providers,” “navigating the healthcare system,” “ability to find good health information,” and “understand health information well enough to know what to do” were statistically significant (P<0.05) (Table 2). The average scores in females were lower than the total average scores were, except in “feeling understood and supported by healthcare providers.” In “actively managing my health,” “social support for health,” and “appraisal of health information” (full marks were 20 points), the highest score was in “social support for health” (males vs. females: 14.73±2.18 vs. 14.56±2.27). The lowest score for males was in “appraisal of health information” (13.24±2.34), and that for females was in “actively managing my health” (13.03±2.38). In “ability to actively engage with healthcare providers,” “ability to find good health information,” and “understand health information well enough to know what to do” (25 points were full marks), the highest score was in “understand health information well enough to know what to do” (males vs. females: 18.84±2.92 vs. 18.27±2.97), and the lowest score was in “ability to actively engage with healthcare providers” (males vs. females: 17.22±3.49 vs. 16.61±3.65). Overall, the worst score was in “feeling understood and supported by healthcare providers” (males vs. females: 8.53±2.39 vs. 8.60±2.31), and the best score was in “understand health information well enough to know what to do” for both males and females.

#### Average score of each scale in different types of residence

In every scale, the average scores in students whose homes were at urban areas were higher than those in rural areas were. The differences in “having sufficient information to manage my health,” “social support for health,” “ability to actively engage with healthcare providers,” “navigating the healthcare system,” “ability to find good health information,” and “understand health information well enough to know what to do” were statistically significant (P<0.05) (Table 2). In “actively managing my health,” “social support for health,” and “appraisal of health information” (20 points were full marks), the highest scores were in “social support for health” (urban areas: 14.72±2.24, rural areas: 14.45±2.26). The lowest score for urban areas was in “actively managing my health” (13.13±2.53) and for rural areas was in “appraisal of health information” (13.03±2.22). We also found that in “ability to actively engage with healthcare providers,” “ability to find good health information,” and “understand health information well enough to know what to do” (25 points were full marks), the lowest score was in “ability to actively engage with healthcare providers” (urban areas: 17.04±3.45, rural areas: 16.39±3.80) and the highest score was in “understand health information well enough to know what to do” (urban areas: 18.73±2.95, rural areas: 18.00±2.96). Overall, the worst score was in “feeling understood and supported by healthcare providers” (urban areas: 8.61±2.33, rural areas: 8.57±2.31). The best score for urban areas was in “understand health information well enough to know what to do” and for rural areas was in “social support for health.”

#### Average score of each scale in different faculties

In every scale, the average scores of engineering students were higher than those of medical/health sciences students were, and the difference was statistically significant (P<0.01) (Table 2). In “actively managing my health,” “social support for health,” and “appraisal of health information” (20 points were full marks), the highest score was in “social support for health” (engineering: 15.31±2.37, medical/health sciences: 14.54±2.23). The lowest score for engineering was in “appraisal of health information” (13.92±2.83) and for medical/health sciences was in “actively managing my health” (13.03±2.36). We found that in “ability to actively engage with healthcare providers,” “ability to find good health information,” and “understand health information well enough to know what to do” (full mark was 25 points), the lowest score was in “ability to actively engage with healthcare providers” (engineering: 18.92±3.57, medical/health sciences: 16.57±3.58) and the highest scores were in “understand health information well enough to know what to do” (engineering: 20.12±2.38, medical/health sciences: 18.25±2.97). Overall, the worst score was in “feeling understood and supported by healthcare providers” (engineering: 9.62±2.62, medical/health sciences: 8.51±2.28) and the best score was in “understand health information well enough to know what to do” for both engineering and medical/health sciences.

### Multiple linear regression to predict influence factors of HLQ scores

As shown in Table 3, we found that in multiple linear regression models (R_adj_^2^ = 0.435, P = 0.000), grade (X1), faculty (X2), depression or anxiety (X3), socioeconomic status (X4), and parent’s highest level of education (X_5_) were the influence factors. Among these, grade, socioeconomic status, and parent’s highest level of education were positively correlated with health literacy:

Y= 114.033+14.302X1−12.810X2−12.013X3+2.742X4+0.970 X5.

**Table 3:** Factors That Influence Health Literacy.

Influence factors	β	SE	T	P
Constant	114.033	5.012	22.752	0.000
Grade	14.302	0.630	22.696	0.000
Faculty	−12.810	1.702	−7.526	0.000
Depression or anxiety	−12.013	2.995	−4.011	0.000
Socioeconomic status	2.742	0.468	5.863	0.000
Parent’s highest level of education	0.970	0.292	3.320	0.001

## Discussion

Health literacy is an important factor that affects health [25]. People with low health literacy face high mortality rates [26, 27], are less knowledgeable about diseases, and have low self-management skills [28–30]. Poor health literacy could also lead to high healthcare costs. The current study found low scores in all domains, and the lowest score recorded was “feeling understood and supported by healthcare providers.” The results indicated that participants who scored low on this domain are unable to engage with doctors and other healthcare providers. They do not have regular healthcare providers and/or have difficulty in trusting healthcare providers as a source of information and/or advice. The highest score, which was still considerably low, was found in “understanding health information well enough to know what to do.” This finding indicated that participants have some problems understanding written health information or instructions about treatments or medications and are unable to read or write sufficiently well to complete medical forms. As grade increased, the average scores of HLQ also increased. This result indicated that school education may play an important role in health literacy, and previous studies have shown that health literacy is closely associated with education [31]. In addition, the average scores of HLQ in males were higher than the scores in females; this finding is different from the monitoring results for health literacy of Chinese residents in 2012 [11]. We found a significant difference (P<0.05) in age, socioeconomic status, and major between males and females. Among those who majored in nursing, 67.3% were females and only 24.8% were males. Different professions have different courses, which may have resulted in higher HLQ average scores in males than females. However, further research is needed to determine the reasons behind this phenomenon. We also found that the average scores of HLQ in urban areas were higher than those in rural areas were because economic development levels and health resource allocations are lower in rural areas than in urban areas. Moreover, we found that the average scores of engineering students were higher than the average scores of medical or health sciences students, which may be due to the different course installations of the two faculties.

Through multiple linear regression models, we found that grade, faculty, depression or anxiety, socioeconomic status, and parent’s highest level of education were the influence factors. In college, as grade increased, the received education also increased. Research has proven that health literacy is closely related to socioeconomic status and education [31]. In addition, Pawlak’s study also showed that low-income level is an important reason for low health literacy, which leads to worse health and higher hospital admission rates [32]. Research has shown that people with low health literacy are more likely to have symptoms of depression [33], which is consistent with our findings. Different faculties also affect health literacy, and these two have a negatively correlated relationship. In our survey, we investigated two faculties, namely, engineering and medical or health sciences. The scores of the engineering faculty were higher than the scores of the medicine or health sciences faculty. This finding may have resulted from the different course installations between the two faculties, and engineering students may have a better understanding of health literacy. Moreover, we still found that parents’ education level was positively correlated with health literacy, which may be because parents with higher education level pay more attention to health and can better guide their children in this area. This suggests the importance of family education to improve the level of children’s health literacy.

Nonetheless, this study also has limitations. First, the cross-sectional survey data do not make direct causal inferences and cannot determine the direction of causality. Second, we found that faculty and health literacy scores are negatively correlated with each other, and the scores of engineering students were higher than the scores of medicine or health sciences students. The engineering faculty only had 89 students and the medicine or health sciences faculty included 1,183 students; thus, the large difference may have affected the establishment of the multiple linear regression models. Third, large differences exist in the numbers of females and males. However, this is a reality in our selected medical university. In addition, 721 participants in the survey are nursing students, who are almost all females. Therefore, we cannot compare differences of males and females in scoring. In addition, we cannot determine whether gender is an impact factor of the score.

## Conclusion

This investigation showed that the health literacy of medical students needs to be improved. Schools should play a leading role and pay more attention to the cultivation of health literacy of students.

## Supporting Information

S1 File Data. (XLS)
